# Oxidative Stress Leads to β-Cell Dysfunction Through Loss of β-Cell Identity

**DOI:** 10.3389/fimmu.2021.690379

**Published:** 2021-11-04

**Authors:** Floris Leenders, Nathalie Groen, Natascha de Graaf, Marten A. Engelse, Ton J. Rabelink, Eelco J. P. de Koning, Françoise Carlotti

**Affiliations:** ^1^ Department of Internal Medicine, Leiden University Medical Center, Leiden, Netherlands; ^2^ Hubrecht Institute, KNAW (Royal Netherlands Academy of Arts and Sciences) and University Medical Center Utrecht, Utrecht, Netherlands

**Keywords:** oxidative stress, beta-cell dysfunction, beta-cell identity, beta-cell dedifferentiation, type 1 diabetes mellitus (T1D)

## Abstract

Pancreatic β-cell failure is a critical event in the onset of both main types of diabetes mellitus but underlying mechanisms are not fully understood. β-cells have low anti-oxidant capacity, making them more susceptible to oxidative stress. In type 1 diabetes (T1D), reactive oxygen species (ROS) are associated with pro-inflammatory conditions at the onset of the disease. Here, we investigated the effects of hydrogen peroxide-induced oxidative stress on human β-cells. We show that primary human β-cell function is decreased. This reduced function is associated with an ER stress response and the shuttling of FOXO1 to the nucleus. Furthermore, oxidative stress leads to loss of β-cell maturity genes MAFA and PDX1, and to a concomitant increase in progenitor marker expression of SOX9 and HES1. Overall, we propose that oxidative stress-induced β-cell failure may result from partial dedifferentiation. Targeting antioxidant mechanisms may preserve functional β-cell mass in early stages of development of T1D.

## Introduction

Type 1 diabetes (T1D) is a chronic autoimmune disease caused by T cell-mediated destruction of pancreatic insulin-producing β-cells (1–3). Autoimmune recognition of β-cell antigens leads to decreased β-cell mass and the subsequent decline of insulin-mediated regulation of glucose levels in the blood eventually results in chronic hyperglycemia and T1D.

Oxidative stress has been implicated in the onset of β-cell failure in T1D (4–6). Plasma levels of oxidative stress markers such as malondialdehyde and protein carbonyl groups are increased upon early onset of T1D and are even higher by early adulthood (7). Oxidative stress occurs when reactive oxygen species (ROS) levels overcome antioxidant defenses. In physiological conditions, ROS, a by-product of mitochondrial metabolism, act as signaling molecules for glucose-stimulated insulin secretion in β-cells. β-cells have been shown to be particularly sensitive to oxidative stress when compared to other islet cell types, as they display low levels of antioxidant enzymes such as superoxide dismutase, glutathione peroxidase, and catalase (8–10).

In recent years, loss of β-cell identity has been proposed as a new mechanism underlying the β-cell failure that is central to the onset and development of diabetes mellitus (11). Alterations in β-cell identity impact their functionality, as indicated by a decreased expression of key β-cell markers such as MAFA, and genes involved in glucose-stimulated insulin secretion such as the glucose transporter Slc2a2 (GLUT2) (12). The concept of β-cell identity loss is strongly supported by experiments performed in murine models and based on lineage-tracing of β-cells. Most of these *in vitro* and *in vivo* experiments involve genetic manipulations (13). Forced deletion of the β-cell-specific transcription factor FOXO1 converts adult murine β-cells into cells with an α-cell, δ-cell or pp-cell phenotype (11). The loss of PDX1, NKX6.1, PAX6 or NKX2.2 leads to β-cells gaining α-cell (14), δ-cell (15), ϵ-cell (16) or polyhormonal cell (17) characteristics, respectively. Additionally, inducing hyperglycemia in mice triggers β-cells to start expressing glucagon (18) or the non-endocrine peptide hormone gastrin (19). Hyperglycemia also leads to β-cell dedifferentiation in mice, as shown by the loss of key genes responsible for β-cell identity and function (11, 16). Data on human β-cell identity loss, on the other hand, remain scarce and mainly descriptive, based on histological analyses performed on donor-derived pancreatic tissue sections. In samples from T1D individuals, there is an increased frequency of hormone-negative endocrine cells (that express none of the islet hormones but that do express the endocrine marker chromogranin A) (20). We reported an eight times increased frequency of insulin-positive cells co-expressing glucagon and a five times increased frequency of NKX6.1-positive, insulin-negative cells co-expressing glucagon in donors with type 2 diabetes compared to the control group (21). We also found an increased proportion of α- and β-cells expressing the mesenchymal protein vimentin in islets from T2D individuals, indicating phenotypic plasticity in the form of an epithelial-to-mesenchymal transition (22). Furthermore, pancreatic sections of T2D donors contain insulin-depleted, degranulated β-cells (23) and show a higher portion of endocrine cells expressing none of the typical endocrine cell markers insulin, glucagon, somatostatin or pancreatic polypeptide (24). Although the concept of β-cell identity loss in diabetes is gaining ground, its underlying mechanisms remain unclear.

Here, we investigate the effects of hydrogen peroxide-induced oxidative stress on human β-cell function and identity to characterize the underlying molecular mechanisms of β-cell failure in diabetes.

## Materials And Methods

### Primary Human Islets and Human β-Cell Line

Pancreata were obtained from cadaveric human organ donors. Human islet isolations from 16 non-diabetic donors (see **Supplemental Table 1**) were performed in the Good Manufacturing Practice facility of our institute (25). Islets were used for research only if they could not be used for clinical purposes and if research consent was present, according to Dutch national laws. Islets with a purity of at least 80% were cultured in regular CMRL 1066 medium (5.5 mmol/L glucose) containing 10% fetal calf serum, 20 mg/mL ciprofloxacin, 50 mg/mL gentamycin, 2 mmol/L L-glutamin, 10 mmol/L HEPES, and 1.2 mg/mL nicotinamide. Islets were maintained in culture at 37°C in 5% CO_2_-humidified atmosphere and medium was refreshed the day after isolation and every two days thereafter.

EndoC-βH1 cells (26) were obtained from Univercell Biosolutions and were cultured in low glucose DMEM supplemented with 5.5 μg/ml human transferrin, 10 mM nicotinamide, 6.7 ng/ml selenit, 2% BSA fraction V, 100 units/ml penicillin, 100 μg/ml streptomycin and 50 μM β-mercaptoethanol. Cells were seeded in pre-coated culture plates containing ECM with fibronectin, maintained in culture at 37°C in 5% CO_2_-humidified atmosphere and passaged once a week.

### Hydrogen Peroxide Treatment

Hydrogen peroxide (H_2_O_2_, Sigma) was diluted in culture medium to prepare a stock solution of 10 M. The stock solution was then further diluted in culture medium to obtain the final concentrations. Human islets or EndoC-βH1 cells were treated with either 50 µM H_2_O_2_ for 24 hours or 200 µM H_2_O_2_ for 90 minutes (with a washing-out period of 22.5 hours afterwards) with read-outs performed 24 hours after the start of the treatment with H_2_O_2_, unless stated otherwise in the figure legends. A schematic overview of the experimental setup is shown in Supplementary **Figure 1A**. For inhibitory experiments, human islets or EndoC-βH1 cells were pre-incubated overnight with tauroursodeoxycholic acid (TUDCA, Millipore) prior to the H_2_O_2_ treatments as shown in **Supplementary Figure 1A**, as well as co-incubated together with H_2_O_2_ for the duration of the treatments. TUDCA was dissolved in water to prepare a stock solution, which was then further diluted in culture medium to obtain the final concentration of 1 mM.

**Figure 1:**
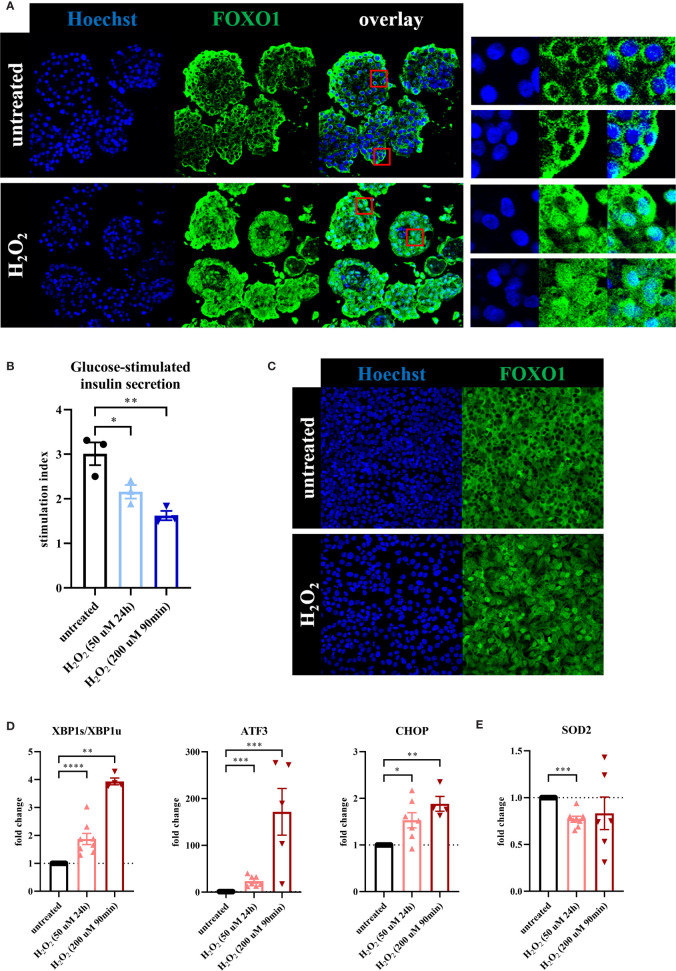
Oxidative stress leads to loss of β-cell function associated with a β-cell stress response. To evaluate the effect of oxidative stress on β-cell function and stress response, primary human islets (blue graphs) or EndoC-βH1 cells (red graphs) were treated with hydrogen peroxide (H_2_O_2_). **(A)** Treatment of human islets with 100 μM H_2_O_2_ for 90 minutes, assessed after 72 hours, leads to shuttling of the transcription factor FOXO1 from its normal cytoplasmic localization to the nucleus, as seen in the magnifications in the panels on the right. Nuclear FOXO1 translocation (as assessed by the overlap of FOXO1 and Hoechst staining) occurred in 94% of cells after H_2_O_2_ treatment, compared to 5% of cells in the untreated condition. **(B)** H_2_O_2_-induced oxidative stress in human islets leads to decreased glucose-stimulated insulin secretion. GSIS was performed 24 hours after the start of the 90min 200 μM H_2_O_2_ treatment or immediately after the 24h 50 μM H_2_O_2_ treatment. **(C)** Daily treatment of EndoC-βH1 cells with 100 μM H_2_O_2_ for 90 minutes, assessed after 72 hours, leads to shuttling of the transcription factor FOXO1 from its normal cytoplasmic localization to the nucleus. **(D)** Oxidative stress induction in EndoC-βH1 cells by treatment with 50 μM H_2_O_2_ for 24 hours and 200 μM H_2_O_2_ for 90 minutes leads to increased mRNA expression levels of the unfolded protein response-related genes XBP1s/XBP1u, ATF3 and CHOP as measured by qPCR. **(E)** Oxidative stress induction in EndoC-βH1 cells by treatment with 50 μM H_2_O_2_ for 24 hours leads to a decreased mRNA expression level of the mitochondrial enzyme SOD2 as measured by qPCR. Data are presented as means ± SEM of fold change over untreated control islets (blue graphs) or EndoC-βH1 cells (red graphs). n = 3-8 donors/batches; each data point represents one donor/batch. *p < 0.05, **p < 0.01, ***p < 0.0005, ****p < 0.0001 *vs.* untreated control islets/EndoC-βH1 cells as determined by an unpaired Student’s t test on the stimulation indices (for the glucose-stimulated insulin secretion data) or a paired Student’s t test on the dCT values (for the qPCR data). Black circles = untreated control islets/EndoC-βH1 cells, upward-pointing triangles = H_2_O_2_-treated islets/EndoC-βH1 cells (50 μM 24h), downward-pointing triangles = H_2_O_2_-treated islets/EndoC-βH1 cells (200 μM 90min). h = hours, min = minutes, H_2_O_2_ = hydrogen peroxide.

### FDA/PI Staining

An FDA/PI staining was performed to assess the viability of human islets or EndoC-βH1 cells after H_2_O_2_ treatment. A staining solution was prepared by diluting an FDA stock solution (5 mg/mL, consisting of fluorescein diacetate (Sigma) and aceton) and a PI stock solution (2 mg/mL, consisting of propidium iodide (Sigma) and PBS) in culture medium without fetal calf serum. Human islets or EndoC-βH1 cells were washed once with PBS and staining solution was added for approximately 5 minutes in the dark at room temperature. Thereafter, human islets or EndoC-βH1 cells were washed with PBS and imaging was done using the EVOS (Invitrogen).

### Glucose-Stimulated Insulin Secretion (GSIS)

Approximately 50 IEQ per well were placed in a 96-well transwell plate. Islets were preincubated for 90 minutes in low (1.67 mmol/L) glucose-containing KRBH (Krebs-Ringer Bicarbonate Hepes) buffer (11.5 mmol/L NaCl, 0.5 mmol/L KCl, 2.4 mmol/L NaHCO_3_, 2.2 mmol/L CaCl_2_, 1 mmol/L MgCl_2_, 20 mmol/L HEPES, and 0.2% human serum albumin) at pH 7.4. Islets were subsequently transferred to low (1.67 mmol/L) glucose-containing buffer for 1 hour and then to high (17 mmol/L) glucose-containing buffer for 1 hour. Insulin secretion was assessed using a human insulin ELISA kit (Mercodia) following the manufacturer’s instructions. In addition, insulin content of the lysed human islets that were used for the glucose-stimulated insulin secretion assay was measured using the same human insulin ELISA kit. To determine basal insulin secretion levels, the insulin secretion was corrected for the amount of DNA present in the lysed human islets that were used for the glucose-stimulated insulin secretion assay. DNA content was measured using a Quant-iT PicoGreen dsDNA assay kit (Thermo Fisher Scientific) following the manufacturer’s instructions.

### Immunofluorescence Microscopy

Human islets and EndoC-βH1 cells were fixed with 4% paraformaldehyde for 10 minutes, permeabilized using 0.5% triton-X for 15 minutes and blocked using goat serum for 15 minutes. Primary and secondary antibodies (Alexa Fluor, Thermo Fisher Scientific) diluted in buffer containing 5% bovine serum albumin were sequentially incubated for 1 hour. After counterstaining with Hoechst (BD), samples were mounted using DABCO-glycerol on microscopy slides and confocal imaging was done using the SP8 WLL (Leica). A primary antibody against FOXO1 (Cell Signaling Technology, 2880) was used. Manual counting was performed using ImageJ to assess the overlap of nuclear FOXO1 and Hoechst.

### RNA Isolation and Quantitative PCR

Human islets or EndoC-βH1 cells were washed in PBS. Total RNA was extracted using (Micro) RNeasy kit (Qiagen) according to the manufacturer’s instructions and the concentration was determined using NanoDrop (Thermo Fisher Scientific). Total RNA was reverse transcribed using M-MLV reverse transcriptase (Invitrogen) and oligo(dT). Quantitative PCR was performed in a CFX system (Bio-Rad). Fold change was calculated using the delta delta CT method with human β-actin or GAPDH as reference gene. Primers used are listed in **Supplemental Table 2**.

### Western Blot

Human islets were washed with cold PBS and lysed using Laemmli sample buffer (60 mmol/l tris pH 6.8, 10% glycerol, 1% SDS, 0.001% blue bromophenol and 5% β-mercaptoethanol). Protein content in the supernatant was quantified using a BCA assay and immunoblotted with antibodies MAFA (Bethyl, A700-067), PDX1 (Abcam, AB47267) and SOX9 (Cell Signaling Technology, 82630S). HRP-conjugated secondary antibodies were used and the signal was developed using enhanced chemiluminescent substrate. The bands were quantified using ImageLab software (BioRad).

### Statistical Analysis

All data are expressed as means ± SEM, unless stated otherwise. For analysis of qPCR data, statistical significance of differences between two groups was determined by a paired or unpaired Student’s t test on the delta CT values calculated from the reference gene (β-actin or GAPDH) and the gene in question. A *P* value below 0.05 was considered statistically significant.

## Results

### Oxidative Stress Leads to Loss of β-Cell Function Associated With a β-Cell Stress Response

To assess the effect of oxidative stress on human β-cell function, we treated primary human islets with 50 μM H_2_O_2_ for 24 hours, or with 200 μM H_2_O_2_ for 90 minutes followed by a wash-out period of 22.5 hours (**Supplementary Figure 1A**). These conditions were chosen because they have been shown to induce an oxidative stress response in pancreatic β-cells with limited cytotoxicity (27–29) and the objective of this study was to investigate the identity of the cells surviving oxidative stress. We monitored the potential toxicity of the H_2_O_2_ treatments on primary human islets with an FDA/PI staining performed at the end of the treatments (**Supplementary Figure 1B**) and relative RNA content was determined as indirect measurement of cell number (**Supplementary Figure 1C**). Although we found a decrease in relative RNA content in the 200 μM H_2_O_2_ condition in particular, we also confirmed a high viability of the remaining cells at t=24h, the main endpoint in this study.

We validated the effect of H_2_O_2_ in human islets by showing a shuttling of the transcription factor FOXO1 from its normal cytoplasmic localization to the nucleus upon H_2_O_2_ treatment (**Figure 1A**), a known adaptation response to oxidative stress (30). Nuclear FOXO1 translocation occurred in 94% of cells after H_2_O_2_ treatment, compared to 5% of cells in the untreated condition. Furthermore, glucose-stimulated insulin secretion (GSIS) displayed a 30% reduction in the 50 μM H_2_O_2_ condition and a 45% decrease after treatment with the 200 μM H_2_O_2_ condition, as compared to the untreated control condition (**Figure 1B**), indicating impaired β-cell function. Basal insulin secretion of the islets that were used for the GSIS assay was not significantly altered after both H_2_O_2_ treatments (**Supplementary Figure 1D**).

In order to evaluate the effect of oxidative stress on β-cells specifically, we used the human β-cell line EndoC-βH1 as a model. H_2_O_2_-induced oxidative stress induced some significant cell death on these cells as reflected by reduced relative RNA content at the end of both treatments (**Supplementary Figure 1F**). Yet, as for human islets, we found a high viability of the remaining cells at t=24h (**Supplementary Figure 1E**). We also observed a shuttling of FOXO1 from the cytoplasm to the nucleus (**Figure 1C**). Furthermore, we found an increased expression of the typical ER stress markers XBP1s/XBP1u, ATF3 and CHOP (**Figure 1D**) that correlated with a reduced expression of the mitochondrial enzyme SOD2 (manganese superoxide dismutase) (**Figure 1E**), which may indicate a failure in the adaptive mechanism to oxidative stress.

Collectively, these data indicate that oxidative stress leads to reduced β-cell function associated with a β-cell stress response.

### Oxidative Stress Leads to Loss of β-Cell Maturity Markers and a Concomitant Increase in Progenitor Marker Expression

**Figure 2:**
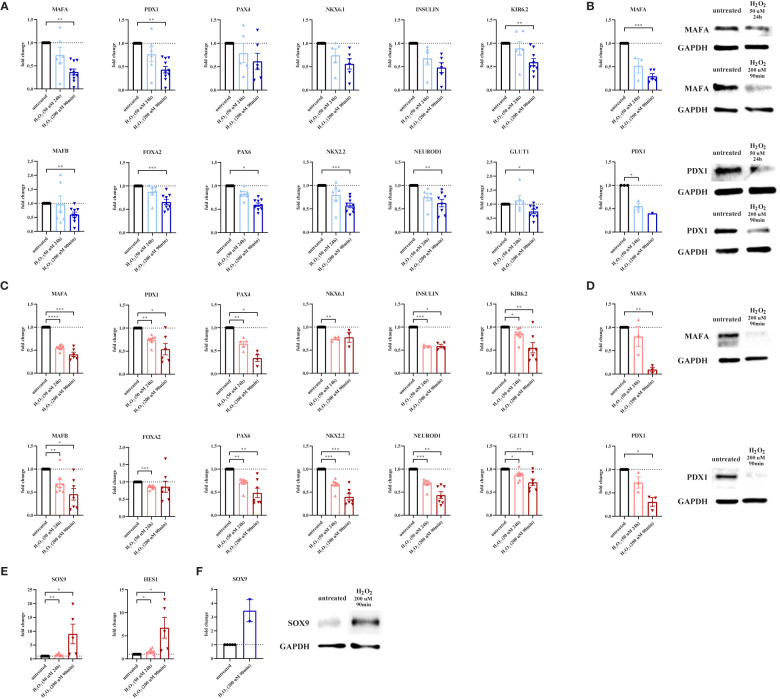
Oxidative stress leads to loss of β-cell maturity markers and a concomitant increase in progenitor marker expression. To evaluate the effect of oxidative stress on β-cell maturity markers and progenitor markers, primary human islets (blue graphs) or EndoC-βH1 cells (red graphs) were treated with hydrogen peroxide (H_2_O_2_). **(A)** Oxidative stress induction in human islets by treatment with 200 μM H_2_O_2_ for 90 minutes leads to decreased mRNA expression levels of the β-cell-specific genes MAFA, PDX1, KIR6.2, MAFB, FOXA2, PAX6, NKX2.2, NEUROD1 and GLUT1 as measured by qPCR. **(B)** The level of β-cell-specific protein MAFA is decreased in human islets upon treatment with 200 μM H_2_O_2_ for 90 minutes as measured by Western blot. **(C)** Oxidative stress induction in EndoC-βH1 cells by treatment with 50 μM H_2_O_2_ for 24 hours and 200 μM H_2_O_2_ for 90 minutes leads to decreased mRNA expression levels of the β-cell-specific genes MAFA, PDX1, PAX4, NKX6.1, insulin, KIR6.2, MAFB, FOXA2, PAX6, NKX2.2, NEUROD1 and GLUT1 as measured by qPCR. **(D)** The level of β-cell-specific proteins MAFA and PDX1 is decreased in EndoC-βH1 cells upon treatment with 200 μM H_2_O_2_ for 90 minutes as measured by Western blot. **(E)** Oxidative stress induction in EndoC-βH1 cells by treatment with 50 μM H_2_O_2_ for 24 hours and 200 μM H_2_O_2_ for 90 minutes leads to increased mRNA expression levels of the progenitor cell-specific genes SOX9 and HES1 as measured by qPCR. **(F)** The level of progenitor cell-specific protein SOX9 is increased in human islets upon treatment with 200 μM H_2_O_2_ for 90 minutes as measured by Western blot. Data are presented as means ± SEM of fold change over untreated control islets (blue graphs) or EndoC-βH1 cells (red graphs). n=1-10 donors/batches; each data point represents one donor/batch. *p < 0.05, **p < 0.01, ***p < 0.0005, ****p < 0.0001 *vs.* untreated control islets/EndoC-βH1 cells as determined by a paired Student’s t test on the dCT values. Black circles = untreated control islets/EndoC-βH1 cells, upward-pointing triangles = H_2_O_2_-treated islets/EndoC-βH1 cells (50 μM 24h), downward-pointing triangles = H_2_O_2_-treated islets/EndoC-βH1 cells (200 μM 90min). h = hours, min = minutes, H_2_O_2_ = hydrogen peroxide.

We next hypothesized that oxidative stress could alter β-cell identity, as a potential mechanism allowing these cells to survive. We first determined the effect of oxidative stress induction on key β-cell genes in primary human islets. At 24 hours after the start of a 90min treatment with H_2_O_2_, gene expression of the maturity marker MAFA was strongly decreased, both at mRNA (**Figure 2A**) and protein level (**Figure 2B**). Similarly, gene and protein expression of the key transcription factor PDX1 was reduced by over 60% (**Figures 2A, B**). A similar trend was observed 24h after treatment with 50 μM H_2_O_2_ (**Figure 2A**), as well as for the gene expression of insulin, of the regulator of β-cell fate PAX4 and of the key β-cell transcription factor NKX6.1 (**Figure 2A**). Other β-cell-related genes that were noticeably decreased after treatment with 200 μM H_2_O_2_ for 90 minutes, were KIR6.2, MAFB, FOXA2, PAX6, NKX2.2 and NEUROD1 (**Figure 2A**). Gene expression of the glucose transporter GLUT1, which plays an important role in β-cell glucose metabolism, was also decreased after treatment with 200 μM H_2_O_2_ for 90 minutes, partly explaining the impaired function shown in **Figure 1A**.

We confirmed these findings in EndoC-βH1 cells, in which gene expression of MAFA, PDX1, PAX4, NKX6.1, insulin, KIR6.2, MAFB, FOXA2, PAX6, NKX2.2, NEUROD1 and GLUT1 was reduced upon H_2_O_2_ treatment (**Figure 2C**). Reduced MAFA and PDX1 expression was also validated at protein level (**Figure 2D**).

Next, we assessed the effect of oxidative stress on endocrine progenitor markers in β-cells. Strikingly, gene expression of SOX9 and HES1 was increased by 50% and 60% after 24h treatment with 50 μM H_2_O_2_, and up to 8- and 6-fold increased after 90min treatment with 200 μM H_2_O_2_, respectively (**Figure 2E**). The increased SOX9 gene expression in EndoC-βH1 cells was confirmed on the protein level in primary human islets (**Figure 2F**).

Altogether, these data show that oxidative stress results in severe alterations in β-cell maturity marker expression, associated with increased expression of progenitor markers, indicating β-cell dedifferentiation as a response to oxidative stress.

### TUDCA Partially Inhibits Oxidative Stress-Induced Detrimental Effects

**Figure 3:**
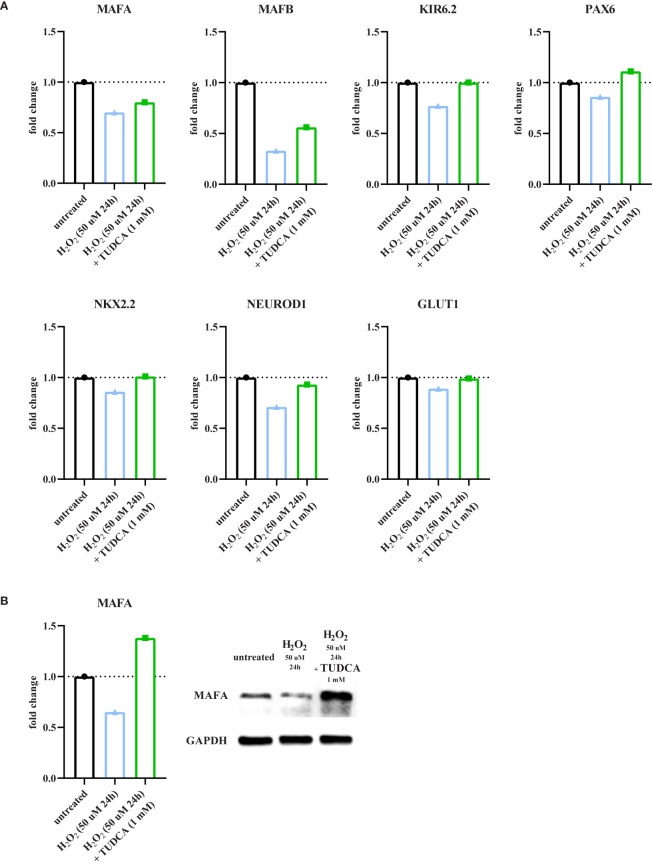
TUDCA partially prevents oxidative stress-induced detrimental effects. To evaluate the effect of tauroursodeoxycholic acid (TUDCA) on oxidative stress-induced detrimental effects, primary human islets were pre-incubated overnight with TUDCA prior to the H_2_O_2_ treatments as shown in **Supplementary Figure 1A**, as well as co-incubated together with H_2_O_2_ for the duration of the treatments. **(A)** TUDCA partially or completely recovers the oxidative stress-induced decreased mRNA expression levels of the genes MAFA, MAFB, KIR6.2, PAX6, NKX2.2, NEUROD1 and GLUT1 in human islets as measured by qPCR. **(B)** The oxidative stress-induced decreased level of the β-cell-specific protein MAFA is recovered by TUDCA in human islets as measured by Western blot. Data are presented as means of technical triplicates as fold change over untreated control islets (blue graphs). n = 1 donor; Black circles = untreated control islets, upward-pointing triangles = H_2_O_2_-treated islets (50 μM 24h), downward-pointing triangles = H_2_O_2_-treated islets (200 μM 90min), green squares = H_2_O_2_ (50 μM 24h or 200 μM 90min) and TUDCA (1 mM) pre- and cotreated islets. h = hours, min = minutes, H_2_O_2_ = hydrogen peroxide, TUDCA = tauroursodeoxycholic acid.

Finally, we hypothesized that oxidative stress-induced detrimental effects on β-cells were partly resulting from ER stress. We targeted the ER stress response by using tauroursodeoxycholic acid (TUDCA), a chemical chaperone that is known to attenuate ER stress and prevent unfolded protein dysfunction (31, 32). Primary human islets were pre-incubated overnight with 1 mM TUDCA prior to the H_2_O_2_ treatments, as well as co-incubated together with H_2_O_2_ for the duration of the treatments. As expected, oxidative stress reduced gene expression of MAFA, MAFB, KIR6.2, PAX6, NKX2.2, NEUROD1 and GLUT1 (**Figure 3A**). Interestingly, this effect was partially or completely prevented by TUDCA treatment (**Figure 3A**). This was confirmed on the protein level for MAFA (**Figure 3B**).

Collectively, these findings are in line with our proposed model that oxidative stress-induced ER stress is a key factor in altered β-cell identity, and therefore function.

## Discussion

Our study demonstrates that oxidative stress leads to loss of β-cell function that is associated with a stress response and evidence of dedifferentiation, as indicated by the loss of β-cell maturity markers and the upregulation of endocrine progenitor markers.

Our finding that β-cell function is decreased upon oxidative stress is supported by studies performed in animal β-cell lines, where the decreased insulin gene expression that follows supraphysiologic concentrations of glucose is prevented by antioxidant treatment, suggesting the involvement of oxidative stress in β-cell failure (33).

We observed that reduced human β-cell function is associated with shuttling of FOXO1 to the nucleus, which has been shown to protect murine β-cells from oxidative stress by increasing the expression of NeuroD and MAFA (34). On the other hand, we found the gene expression of the mitochondrial enzyme SOD2 to be downregulated upon H_2_O_2_ treatment. Since SOD2 functions as an antioxidant, its reduced expression could lead to excessive ROS, which is known to suppress β-cell mitochondrial activity and other components of the insulin secretion pathway, thereby leading to β-cell dysfunction (35–37).

Oxidative stress-induced altered β-cell function was also associated with an ER stress response, as shown by the upregulation of the ER-stress-related genes XBP1s/u (38), ATF3 (39) and CHOP (40). Activation of the unfolded protein response (UPR) is involved in the preservation of β-cell survival and function (41). However, in case of persistent or severe ER stress as in T1D, UPR activation leads to the opposite cell fate, i.e. β-cell dysfunction and death (42–44). In our study, both the FOXO1 shuttling and the initial ER stress response upon H_2_O_2_ treatment may represent attempts to protect β-cells from oxidative stress-induced damages.

Oxidative stress leads to loss of the maturity genes MAFA and PDX1 in human β-cells. We recently described a similar adaptation in primary human islets in a model of drug-induced diabetes (45). In addition, these findings are in line with earlier studies in rodent β-cell lines or islets that link oxidative stress to alterations in β-cell identity, through reduced expression of the key β-cell transcription factors PDX1, NKX6.1 and MAFA (46–49). Additionally, reduced nuclear MAFA expression found in islets of diabetic db/db mice is restored by overexpression of the antioxidant enzyme endogenous glutathione peroxidase-1 (GPX1) (49, 50). Likewise, decreased nuclear expression of PDX1 and MAFA seen in islets of diabetic ZDF rats is prevented by treatment with ebselen, a GPX mimetic (51). Most of these studies were performed in rodent islets or cell lines, whereas our study confirms the link between oxidative stress and the decrease in maturity markers in primary human β-cells. Besides MAFA and PDX1, gene expression of other important regulators of β-cell maturity and identity, such as MAFB (52), PAX6 (16), NKX2.2 (53) and NEUROD1 (54) were also decreased upon H_2_O_2_ treatment, further indicating the link between oxidative stress and altered β-cell identity. Decreased expression of key regulators of glucose sensing and insulin secretion, such as GLUT1 (55), FOXA2 (56) and KIR6.2 (57) after H_2_O_2_ treatment could explain the impaired β-cell function upon oxidative stress.

In parallel to the downregulation of key β-cell genes, we observed a concomitant increase in SOX9 and HES1 expression in EndoC-βH1cells. Increased progenitor cell marker expression (alongside decreased β-cell markers) was previously shown in FGF2- and viral infection-induced dedifferentiation models in EndoC-βH1 cells (58, 59). Similarly, upregulated progenitor markers have been shown in rat pancreatic islets treated with hydrogen peroxide, as seen by the increased expression of C-MYC (60), a transcription factor known to inhibit β-cell differentiation (61, 62).

The decreased expression of β-cell maturity genes and the increased expression of progenitor cell markers could indicate that oxidative stress-induced β-cell failure may result from partial dedifferentiation. It is proposed that dedifferentiation might be a way for β-cells to escape from immune-mediated destruction. A study performed in the NOD mouse model indicates that under inflammatory stress, a subpopulation of β-cells decreases its characteristics of mature β-cells while displaying increased stemness-like features to escape from T-cell-mediated death (63). Furthermore, Lee et al. reported that the loss of β-cell maturity genes (induced by β-cell-specific IRE1α deletion) prevents insulitis, the autoimmune destruction of β-cells and therefore the development of diabetes in NOD mice (64).

We observed that the chemical chaperone TUDCA partially prevents the decrease of β-cell markers such as MAFA, MAFB and PAX6 upon oxidative stress. TUDCA is known to alleviate ER stress in β-cells (65, 66). Additionally, TUDCA has been shown to have anti-oxidant capacities in certain neurological disorders (67, 68). Our findings indicate that oxidative stress-induced ER stress could be a key factor in altered β-cell identity.

In this study, we focused specifically on human β-cells. The impact of oxidative stress on human α-cell identity and function remains to be elucidated, which could potentially be a future focus area. Overall, we propose that oxidative stress-induced β-cell failure may result from partial dedifferentiation, which may be an adaptive mechanism for cells to survive. Targeting antioxidant mechanisms could be an important step in preserving functional β-cell mass in T1D.

## Data Availability Statement

The original contributions presented in the study are included in the article/**Supplementary Material**. Further inquiries can be directed to the corresponding author.

## Ethics Statement

Ethical review and approval was not required for the study on human participants in accordance with the local legislation and institutional requirements. Written informed consent for participation was not required for this study in accordance with the national legislation and the institutional requirements.

## Author Contributions

FL designed and performed the experiments and wrote the manuscript. NG, and NdG performed the experiments. ME and TR provided infrastructure for human islet isolation. FC and EdK supervised the project. FC acquired the funding for the project and wrote the manuscript. All authors contributed to the article and approved the submitted version.

## Funding

Our lab is supported by grants from JDRF, the Dutch Diabetes Research Foundation and the DON Foundation.

## Conflict of Interest

The authors declare that the research was conducted in the absence of any commercial or financial relationships that could be construed as a potential conflict of interest.

## Publisher’s Note

All claims expressed in this article are solely those of the authors and do not necessarily represent those of their affiliated organizations, or those of the publisher, the editors and the reviewers. Any product that may be evaluated in this article, or claim that may be made by its manufacturer, is not guaranteed or endorsed by the publisher.

## Supplementary Material

Supplementary Figure 1 Schematic overview of the experimental setup and validation of the H_2_O_2_ treatments. **(A)** Primary human islets or EndoC-βH1 cells were treated with 50 μM H_2_O_2_ for 24 hours or with 200 μM H_2_O_2_ for 90 minutes. After 90 minutes (t = 90min), the 200 μM H_2_O_2_ was washed away and new medium without H_2_O_2_ was added to the islets or cells. At 24 hours after the start of both treatments (t = 24h), all conditions were harvested for the readouts. **(B)** Viability of human islets after H_2_O_2_ treatments was assessed at t = 24h by FDA/PI staining. **(C)** Relative RNA content of human islets after H_2_O_2_ treatments was measured at t = 24h. **(D)** The basal insulin secretion levels of the islets that were used in the glucose-stimulated insulin secretion assay are unaltered after both H_2_O_2_ treatments. **(E)** Viability of EndoC-βH1 cells after H_2_O_2_ treatments was assessed at t = 24h by FDA/PI staining. **(F)** Relative RNA content of EndoC-βH1 cells after H_2_O_2_ treatments was measured at t=24h. Data are presented as means ± SEM over untreated control islets (blue graphs) or EndoC-βH1 cells (red graphs). n = 3-12 donors/batches; each data point represents one donor/batch. **p < 0.01, ****p < 0.0001 *vs.* untreated control islets/EndoC-βH1 cells as determined by an unpaired Student’s t test on the RNA concentrations. Black circles = untreated control islets/EndoC-βH1 cells, upward-pointing triangles = H_2_O_2_-treated islets/EndoC-βH1 cells (50 μM 24h), downward-pointing triangles = H_2_O_2_-treated islets/EndoC-βH1 cells (200 μM 90min). h = hours, min = minutes, H_2_O_2_ = hydrogen peroxide, FDA = fluorescein diacetate, PI = propidium iodide.
