# Supplementary material for: Oxidative Stress Leads to β-Cell Dysfunction Through Loss of β-Cell Identity
Source: Front Immunol. 2021 Nov 4;12:690379. doi: 10.3389/fimmu.2021.690379 (PMC8601632; doi:10.3389/fimmu.2021.690379)
Supplement: Supplementary file 2 [file DataSheet_1.docx]

**Supplemental Material**

**Supplemental Table 1: list of islet preparations**

| **Unique identifier** | **Donor age (years)** | **Donor sex (M/F)** | **Donor BMI (kg/m^2^)** | **Cause of death** | **Estimated purity (%)** |
| --- | --- | --- | --- | --- | --- |
| P611 | 70 | M | 30 | SAB | 95 |
| R145 | 71 | F | 25 | CVA | 99 |
| R147 | 56 | F | 37 | euthanasia | 85 |
| R148 | 62 | M | 28 | cardiac arrest | 92 |
| R155 | 62 | M | 24 | SAB | 95 |
| R159 | 68 | M | 25 | CVA | 98 |
| R161 | 48 | F | 33 | cardiac arrest | 97 |
| R162 | 74 | M | 24 | CVA | 99 |
| R163 | 55 | F | 22 | cardiac arrest | 92 |
| R165 | 40 | M | 19 | cardiac arrest | 99 |
| R167 | 56 | M | 24 | SAB | 89 |
| R171 | 72 | M | 19 | cardiac arrest | 90 |
| R172 | 71 | M | 25 | CVA | 98 |
| R181 | 73 | F | 23 | trauma | 92 |
| R186 | 70 | M | 25 | SAB | 80 |
| R197 | 73 | M | 25 | CVA | 95 |

**Supplemental Table 2: list of qPCR primers**

| **Gene** | **Primer sequence** |
| --- | --- |
| β-ACTIN | F: 5’-TGCGTGACATTAAGGAGAAG-3’  R: 5’-TGAAGGTAGTTTCGTGGATG-3’ |
| GAPDH | F: 5’-GGAAGCTTGTCATCAATGG-3’  R: 5’-TGATGATCTTGAGGCTGTTG-3’ |
| SOD2 | F: 5’-GGAAGCCATCAAACGTGACT-3’  R: 5’-CTGATTTGGACAAGCAGCAA-3’ |
| XBP1s | F: 5’-CTGAGTCCGCAGCAGGTG-3’  R: 5’-GAGATGTTCTGGAGGGGTGA-3’ |
| XBP1u | F: 5’-GGAGTTAAGACAGCGCTTGG-3’  R: 5’-CTGCAGAGGTGCACGTAGTC-3’ |
| ATF3 | F: 5’-GTGCCGAAACAAGAAGAAGG-3’  R: 5’-TCTGAGCCTTCAGTTCAGCA-3’ |
| CHOP | F: 5’-GACCTGCAAGAGGTCCTGTC-3’  R: 5’-CTCCTCCTCAGTCAGCCAAG-3’ |
| MAFA | F: 5’-AGTCCTGCCGCTTCAAG-3’  R: 5’-ACAGGTCCCGCTCTTTGG-3’ |
| PDX1 | F: 5’-CCATGGATGAAGTCTACCAAAGCT-3’  R: 5’-CGTGAGATGTACTTGTTGAATAGGAACT-3’ |
| PAX4 | F: 5’-AGCAGAGGCACTGGAGAAAGAGTT-3’  R: 5’-CAGCTGCATTTCCCACTTGAGCTT-3’ |
| NKX6.1 | F: 5’-CTGGCCTGTACCCCTCATCA -3’  R: 5’-CTTCCCGTCTTTGTCCAACAA-3’ |
| INSULIN | F: 5’-AAGAGGCCATCAAGCAGATCA-3’  R: 5’-CAGGAGGCGCATCCACA-3’ |
| SOX9 | F: 5’-AGTACCCGCACTTGCACAAC-3’  R: 5’-ACTTGTAATCCGGGTGGTCCTT-3’ |
| HES1 | F: 5’-CCAAAGACAGCATCTGAGCA-3’  R: 5’-CATTGATCTGGGTCATGCAG-3’ |
| C-MYC | F: 5’-TCTCTGAAAGGCTCTCCTTG-3’  R: 5’-CCTGTTGGTGAAGCTAACG-3’ |
| KIR6.2 | F: 5’-GGAGGTAAGGAAGAGTCTGGT-3’  R: 5’-GGGCTTCCCCCATCGGA-3’ |
| MAFB | F: 5’-GCCAAACCGCATAGAGAAC-3’  R: 5’-GGGGATAAGGGAAGGAAAG-3’ |
| FOXA2 | F: 5’-GGGAGCGGTGAAGATGGA-3’  R: 5’-TCATGTTGCTCACGGAGGAGTA-3’ |
| PAX6 | F: 5’-TGGGCAGGTATTACGAGACTG-3’  R: 5’-ACTCCCGCTTATACTGGGCTA-3’ |
| NKX2.2 | F: 5’-CCTTCTACGACAGCAGCGACA-3’  R: 5’-AGACCGTGCAGGGAGTACTGA-3’ |
| NEUROD1 | F: 5’-TGAGACTATCACTGCTCAGG-3’  R: 5’-CACTCTCGCTGTACGATTTG-3’ |
| GLUT1 | F: 5’-TATGTGGAGCAACTGTGTGGT-3’  R: 5’-TCCGGCCTTTAGTCTCAGGA-3’ |
